# *Porphyromonas gingivalis* as an uncommon cause of intracranial abscesses

**DOI:** 10.1590/0037-8682-0370-2020

**Published:** 2021-03-08

**Authors:** Bruno Niemeyer de Freitas Ribeiro, Edson Marchiori

**Affiliations:** 1 Instituto Estadual do Cérebro Paulo Niemeyer, Departamento de Radiologia, Rio de Janeiro, RJ, Brasil.; 2 Labs A+/Grupo Fleury, Rio de Janeiro, RJ, Brasil.; 3 Universidade Federal do Rio de Janeiro, Departamento de Radiologia, Rio de Janeiro, RJ, Brasil.

**FIGURE 1:**
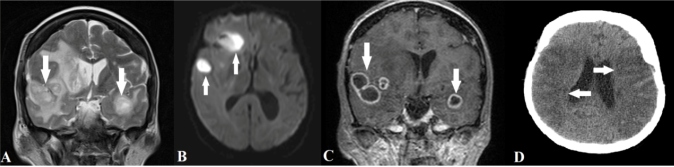
T2-weighted **(A)**, diffusion-weighted **(B)**, and gadolinium-enhanced T1-weighted magnetic resonance images **(C)** showing multiple hyperintense lesions, with restricted diffusibility and peripheral enhancement affecting the bilateral temporal and right frontal lobes (white arrows), suggesting abscesses; brain computed tomography **(D)** revealing an ischemic vascular insult in the territory of the middle cerebral arteries (white arrows).

A 66-year-old woman presented with an approximately 15-day history of headache and no associated symptoms. Her complete blood counts were normal, and blood and urine cultures were negative. Brain magnetic resonance imaging showed multiple lesions in the bilateral temporal and right frontal lobes with T2 hyperintensity, restricted diffusibility, and peripheral enhancement, suggesting abscesses (Figure 1A-C). *Porphyromonas gingivalis* was confirmed as the etiological agent via biopsy. Subsequently, the family members reported that the patient had undergone a dental procedure 1 month before symptom onset. Because of a delayed surgical approach, the lesions progressed and the patient developed an ischemic vascular insult in the territory of the middle cerebral arteries (Figure 1D), probably related to vasculitis secondary to the adjacent inflammatory/infectious process.

Oral cavity pathogens may reach the brain in various ways, such as direct contiguous spread (notably from the maxillary teeth), systemic hematogenous bacteremia (spontaneous or following invasive oral procedures, the most important pathophysiological mechanism), and direct venous drainage^1^ ^-^ ^3^. Most cerebral abscesses associated with oral infection are polymicrobial; most patients present with low levels of oral hygiene, poor periodontal conditions, and chronic infections with endodontic or periodontal lesions affecting multiple teeth^1^ ^-^ ^3^.

Intracranial abscesses are treated by surgical drainage combined with antibiotic therapy (ceftriaxone and metronidazole)^1^ ^-^ ^2^. The infected oral areas must be carefully and systematically identified. Surgical procedures are preferred over restorative procedures such as endodontic treatments^1^ ^-^ ^2^. 

Dental foci should always be considered in the evaluation and treatment of intracranial abscesses of unknown origin.
